# Ramp-Creep Ultrasound Viscoelastography for Measuring Viscoelastic Parameters of Materials

**DOI:** 10.3390/ma13163593

**Published:** 2020-08-14

**Authors:** Che-Yu Lin

**Affiliations:** Institute of Applied Mechanics, College of Engineering, National Taiwan University, No. 1, Sec. 4, Roosevelt Road, Taipei 10617, Taiwan; cheyu@ntu.edu.tw; Tel.: +886-2-3366-5653

**Keywords:** elastography, elasticity, stiffness, viscoelasticity, mechanical properties, biomaterials, material characterization

## Abstract

Several ultrasound-based methods have been developed to evaluate the viscoelastic properties of materials. The purpose of this study is to introduce a novel viscoelastography method based on ultrasound acoustic radiation force for measuring the parameters relevant to the viscoelastic properties of materials, named ramp-creep ultrasound viscoelastography (RC viscoelastography). RC viscoelastography uses two different ultrasound excitation modes to cause ramp and creep strain responses in the material. By combining and analyzing the information obtained from these two modes of excitation, the viscoelastic parameters of the material can be quantitatively evaluated. Finite element computer simulation demonstrated that RC viscoelastography can accurately evaluate the viscoelastic parameters of the material, including the relaxation and creep time constants as well as the ratio of viscous fluids to solids in the material, except for the region near the top surface of the material. The novelty of RC viscoelastography is that there is no need to know the magnitude of acoustic radiation force and induced stress in the material in order to evaluate the viscoelastic parameters. In the future, experiments are necessary to test the performance of RC viscoelastography in real biomaterials and biological tissues.

## 1. Introduction

Ultrasound elastography can noninvasively render images portraying information relevant to the stiffness of materials using ultrasound imaging [1,2]. Ultrasound elastography is believed to be a promising clinical tool for the diagnosis of pathological conditions and for the evaluation of prognosis. For example, it is useful for the staging of liver fibrosis since the fibrotic liver is stiffer than normal liver [3,4,5,6], or can be used to diagnose tendinopathy due to the softened feature of symptomatic tendons [7,8]. It can also be a useful research tool for evaluating the status of biomaterials during the development process, providing mechanical information relevant to cellular mechanobiology [9].

Ultrasound elastography relies on the application of a mechanical excitation to stimulate the material, and then uses ultrasound imaging to monitor the response of the material (i.e., the resulting deformation or strain) [2,10,11]. The information about the excitation and response of the material is used to quantitatively evaluate the information relevant to the stiffness of the material [2,10,11]. Several groups have developed different ultrasound elastography methods, and these can be classified according to the source of excitation used to stimulate the tissue [2,10,11]. For example, in strain elastography, the operator uses a transducer to manually compress the material in order to create an image showing the distribution of strain [12,13,14]. In imaging based on acoustic radiation force, the transducer applies an impulse-like acoustic radiation force excitation to deform the material, and the distribution of displacement can be displayed [15,16]. Shear wave elastography uses acoustic radiation force excitations to generate shear waves which propagate through the material. The speed of shear waves is then measured using ultrasound imaging, and an image showing the distribution of the shear wave speed is created. Since the shear wave speed is proportional to the stiffness of the material, an image showing the map of the stiffness can be rendered [17,18]. In addition to the use of acoustic radiation force, shear waves can also be created by using a mechanical vibrator to externally vibrate the surface of the material [19,20,21,22]. All of the abovementioned methods can provide measures related to information relevant to the stiffness of materials.

Biological tissues and biomaterials are all viscoelastic. Several ultrasound-based methods have been developed to quantitatively evaluate the viscoelastic properties of materials [2,10,11], such as vibro-acoustography [23,24,25], shear wave dispersion ultrasound vibrometry [26,27], shear wave spectroscopy [28], monitored steady-state excitation and recovery [29], multimode ultrasound viscoelastography [30,31], a force-independent method [32], and a model-independent method [33]. The common feature shared by these methods is that they use acoustic radiation force as the source of excitation, although they have their own specific designs and characteristics.

Some groups have developed ultrasound techniques for evaluating the viscoelastic properties of materials based on monitoring the creep response [29,30,31]. In these kinds of methods, the material’s creep curve (increasing strain over time, recorded by ultrasound imaging) is obtained by applying a step force of constant magnitude to the material. The step force is applied by acoustic radiation force using focused ultrasound pulses. The viscoelastic properties of the material can then be evaluated by using a viscoelastic model to curve fit the creep curve. The main limitation of these kinds of methods is that the magnitude of acoustic radiation force is generally unknown due to the unknown absorption coefficient and speed of sound in the material [29,33,34]. Hence, curve fitting may not be able to provide an accurate evaluation of the viscoelastic properties of the material, since the magnitude of acoustic radiation force and induced stress in the material are required for the curve-fitting process.

The purpose of the present study is to introduce a novel viscoelastography method based on ultrasound acoustic radiation force for the noninvasive measurement of the parameters relevant to the viscoelastic properties of a material. The proposed method is named ramp-creep ultrasound viscoelastography (RC viscoelastography), since it uses two different ultrasound excitation modes to cause ramp and creep strain responses in the material. By combining and analyzing the information obtained from these two modes of excitation, the material’s viscoelastic parameters (the relaxation and creep time constants, as well as the ratio of viscous fluids to solids in the material) can be quantitatively evaluated without needing to know the magnitude of acoustic radiation force and induced stress in the material. In this paper, the principle of RC viscoelastography is presented in detail, and its performance and validity are investigated using finite element (FE) computer simulation.

## 2. Materials and Methods

### 2.1. Brief Description of How RC Viscoelastography Works

RC viscoelastography uses two modes, “ramp mode” and “creep mode”, separately and sequentially to excite the material. By combining and analyzing the information obtained from the two modes, the viscoelastic parameters (the relaxation and creep time constants as well as the ratio of viscous fluids to solids in the material) of the material can be quantitatively evaluated without knowing the distribution and magnitude of acoustic radiation force and induced stress in the material.

In the ramp mode, the system applies an acoustic radiation force of linearly increasing magnitude to excite the material. Once the measurement is complete, the acoustic radiation force is released. Following the ramp mode, the creep mode is then used to excite the material by applying an acoustic radiation force of constant magnitude in order to create a creep response.

In each mode, the strain of each element in the material is recorded by ultrasound imaging. The data (strain versus time curves) obtained from the two modes are used to quantitatively evaluate the viscoelastic parameters. The principle of RC viscoelastography and the details of each mode are described in the next section.

**Figure 1:**
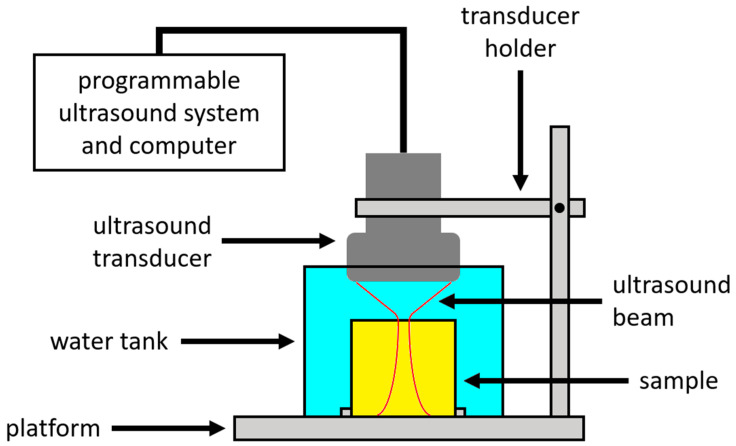
In the future experiment, the experimental setup will consist of a programmable ultrasound system with a phased array ultrasound transducer. The sample is secured on the bottom of the water tank.

In the future experiment, the experimental setup will consist of a programmable ultrasound system (such as the Vantage ultrasound system from Verasonics, Inc., Kirkland, WA, USA, or the Prodigy ultrasound system from S-Sharp Corporation, New Taipei City, Taiwan) with a phased array ultrasound transducer, as shown in Figure 1. The transducer is used to apply acoustic radiation force excitations to induce strains in the sample and to apply imaging beams for the detection of strains. The transducer is fixed by a mechanical holder while its location can be controlled to target the sample. The sample is secured on the bottom of the water tank. The transducer does not have to be in contact with the sample, but the location of the transducer must be carefully adjusted such that the focus of the acoustic radiation force excitation is positioned on the top surface of the sample. The programmable ultrasound system equips the functional components (transmission, reception, process, analysis and display of ultrasound signals, etc.) needed for the development of an ultrasound technique, provides the flexibility to define each of the ultrasound parameters using programming language, and is expected to implement the design of RC viscoelastography. Gelatin gels are expected to be used as samples in the initial experiment for validating the proposed RC viscoelastography method. The viscoelastic properties of gelatin gels measured by a material testing system will serve as the gold standard and compared to those measured by RC viscoelastography. If they are close to each other (i.e., if the difference between them is less than a prescribed level), the validity of RC viscoelastography can be justified. It must be noted that the strain of the sample must be small (small enough that the sample’s mechanical behavior is linear) during the measurement by the material testing system, or the result may not be comparable to that measured by RC viscoelastography, as the strain induced by RC viscoelastography is very small.

### 2.2. Principle of RC Viscoelastography

#### 2.2.1. Linear Viscoelastic Model Used in the Development of the Principle of RC Viscoelastography

**Figure 2:**
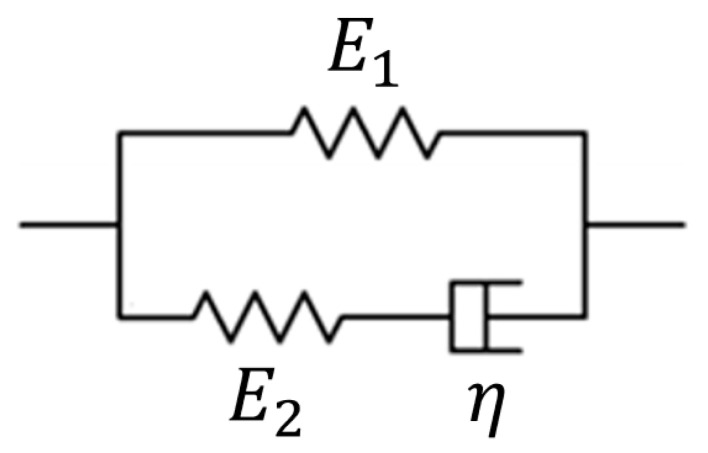
Maxwell form of the standard linear solid model used for describing the viscoelastic behaviors of the material and for developing the principle of ramp-creep ultrasound viscoelastography (RC viscoelastography).

In the present study, the Maxwell form of the standard linear solid model (Figure 2) is used to describe the viscoelastic behaviors of the material. The governing equation for the Maxwell form of the standard linear solid model is:

(1) $$\sigma +\tau_{R}\dot{\sigma }=E_{1}\left(\varepsilon +\tau_{C}\dot{\varepsilon }\right)$$

where $\tau_{R}=\eta /E_{2}$ and $\tau_{C}=\eta \left(E_{1}+E_{2}\right)/E_{1}E_{2}$ are the relaxation and creep time constants, respectively.$\;E_{1}$, $E_{2}$, and η are parameters relevant to the viscoelastic properties of the material. σ is the magnitude of the stress (hereafter referred to as “stress” for brevity) of an element in the material. ε is the magnitude of the strain (hereafter referred to as “strain” for brevity) of that material element.

It has been reported that [35], in the standard linear solid model, there is a physical parameter g defined as:

(2) $$g=1-\frac{\tau_{R}}{\tau_{C}}=\frac{E_{2}}{E_{1}+E_{2}}$$

where g is a real number between 0 and 1. The physical meaning of g is associated with the ratio of viscous fluids to solids in a viscoelastic solid [35].

The relaxation time constant $\tau_{R}$, creep time constant $\tau_{C}$, and g are three parameters relevant to the viscoelastic properties of the material. The capability of the proposed RC viscoelastography described in the present study is to quantitatively evaluate these three parameters.

#### 2.2.2. Ramp Mode

In the ramp mode, the transducer uses impulsive, high-intensity, focused ultrasound pulses to generate an acoustic radiation force of linearly increasing magnitude to induce strains in the material. This acoustic radiation force can be generated by sweeping and increasing the operating frequency of the transducer (i.e., the frequency of the input signal to drive the transducer) linearly with time from an initial frequency (lower than the center frequency of the transducer) until the center frequency is approached. Meanwhile, the voltage for exciting the transducer is kept constant. During this acoustic radiation force excitation, the induced stress of an element in the material also increases linearly with time. Let the stress of an element in the material σ be equal to $\sigma_{0}+rt$ as an increasing linear function with time. r is the rate of increase of σ. Generally, r is not equal to but proportional to the linear rate of increase of the operating frequency of the transducer. r is unknown. $\sigma_{0}$ is the initial increase of the stress immediately following the application of the acoustic radiation force, and is unknown. Substitute σ into Equation (1):

(3) $$\sigma_{0}+rt+\tau_{R}r=E_{1}\left(\varepsilon +\tau_{C}\dot{\varepsilon }\right)$$

If the initial condition is $\varepsilon \left(0\right)=\varepsilon_{0}$, the analytical solution of Equation (3) is:

(4) $$\varepsilon \left(t\right)=\left[\varepsilon_{0}-\frac{\left(\tau_{R}-\tau_{C}\right)r+\sigma_{0}}{E_{1}}\right]e^{-\frac{1}{\tau_{C}}t}+\frac{r}{E_{1}}t+\frac{\left(\tau_{R}-\tau_{C}\right)r+\sigma_{0}}{E_{1}}$$

**Figure 3:**
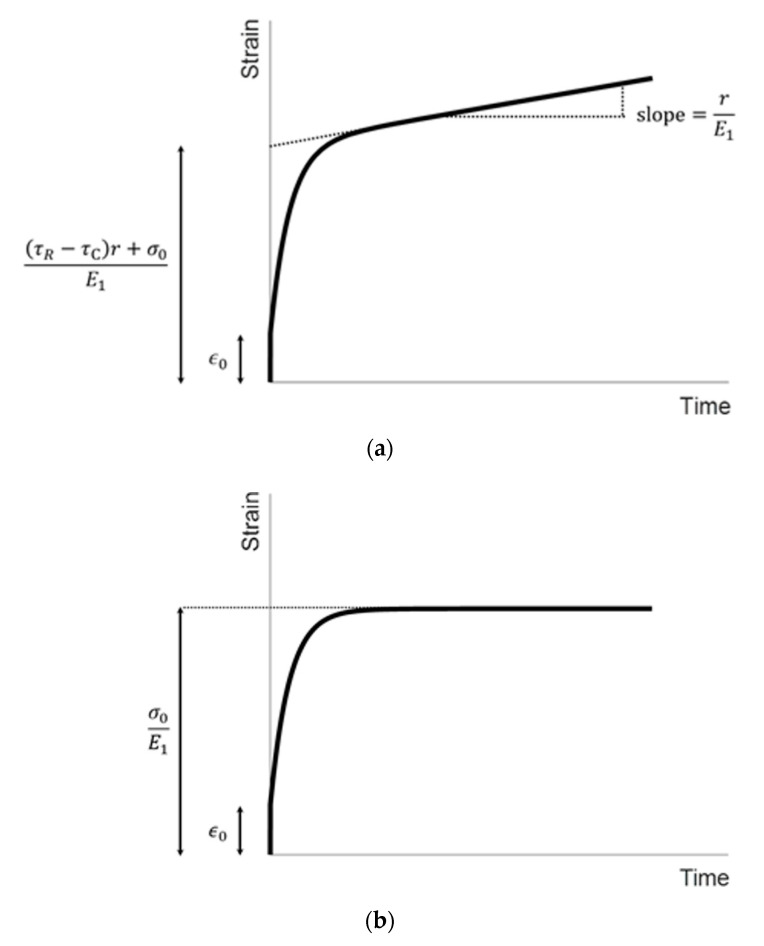
Illustration of the strain responses in the ramp mode (**a**) and creep mode (**b**).

Equation (4) describes the strain response (i.e., the strain versus time) of an element in the material during the application of the ramp acoustic radiation force excitation (Figure 3a). The first term on the right side of Equation (4) describes the transient state, while the second and third terms together describe the steady state. $r/E_{1}$ and $\left[\left(\tau_{R}-\tau_{C}\right)r+\sigma_{0}\right]/E_{1}$ denote the slope of the steady state and the intercept of the steady state on the ε axis, respectively (Figure 3a), and the values of these two can be directly obtained from experimental data. $\varepsilon_{0}$ is the initial increase of the strain immediately following the application of the acoustic radiation force, and can be directly obtained from experimental data (Figure 3a).

#### 2.2.3. Creep Mode

Following the ramp mode, the creep mode is then used to excite the material in order to induce a creep response by applying an acoustic radiation force of constant magnitude.

In the creep mode, the operating frequency of the transducer and the voltage for exciting the transducer are kept as constant. Therefore, the magnitude of acoustic radiation force and induced stress of an element in the material should be constant as well. The operating frequency is set as the center frequency. Let σ equal to $\sigma_{0}$ and substitute it into Equation (1):

(5) $$\sigma_{0}=E_{1}\left(\varepsilon +\tau_{C}\dot{\varepsilon }\right)$$

If the initial condition is $\varepsilon \left(0\right)=\varepsilon_{0}$, the analytical solution of Equation (5) is:

(6) $$\varepsilon \left(t\right)=\left(\varepsilon_{0}-\frac{\sigma_{0}}{E_{1}}\right)e^{-\frac{1}{\tau_{C}}t}+\frac{\sigma_{0}}{E_{1}}$$

Equation (6) describes the creep response of an element in the material during the application of the constant acoustic radiation force excitation (Figure 3b). $\sigma_{0}$ is the same as that in the ramp mode, and is the initial increase of the stress immediately following the application of the acoustic radiation force. $\sigma_{0}$ is unknown. $\varepsilon_{0}$ is the same as that in the ramp mode, and is the initial increase of the strain immediately following the application of the acoustic radiation force. $\sigma_{0}/E_{1}$ is the equilibrium constant of ε(t) at longer times during creep. $\sigma_{0}/E_{1}$ is a known constant since it can be directly obtained from experimental data (Figure 3b). $\tau_{C}$ can be obtained by using Equation (6) to curve fit the creep curve.

#### 2.2.4. Evaluation of the Viscoelastic Properties

The viscoelastic parameters of the material can be quantitatively evaluated as described below, by using the three known values obtained from the ramp mode, $\varepsilon_{0}$, $r/E_{1}$, and $\left[\left(\tau_{R}-\tau_{C}\right)r+\sigma_{0}\right]/E_{1}$, and the two known values obtained from the creep mode, $\sigma_{0}/E_{1}$ and $\tau_{C}$.

$\tau_{R}$ can be evaluated by substituting $r/E_{1}$, $\sigma_{0}/E_{1},$ and $\tau_{C}$ into $\left[\left(\tau_{R}-\tau_{C}\right)r+\sigma_{0}\right]/E_{1}$.

g, a parameter associated with the ratio of viscous fluids to solids in a viscoelastic solid, can be obtained by substituting $\tau_{R}$ and $\tau_{C}$ into Equation (2).

Consequently, three parameters relevant to the viscoelastic properties of the material (i.e., $\tau_{R}$, $\tau_{C}$ and g) of every element in the material can be quantitatively evaluated without knowing the distribution and magnitude of acoustic radiation force and induced stress in the material.

### 2.3. Setting for the Computer Simulation

#### 2.3.1. Basic Setting of the Computer Simulation

**Figure 4:**
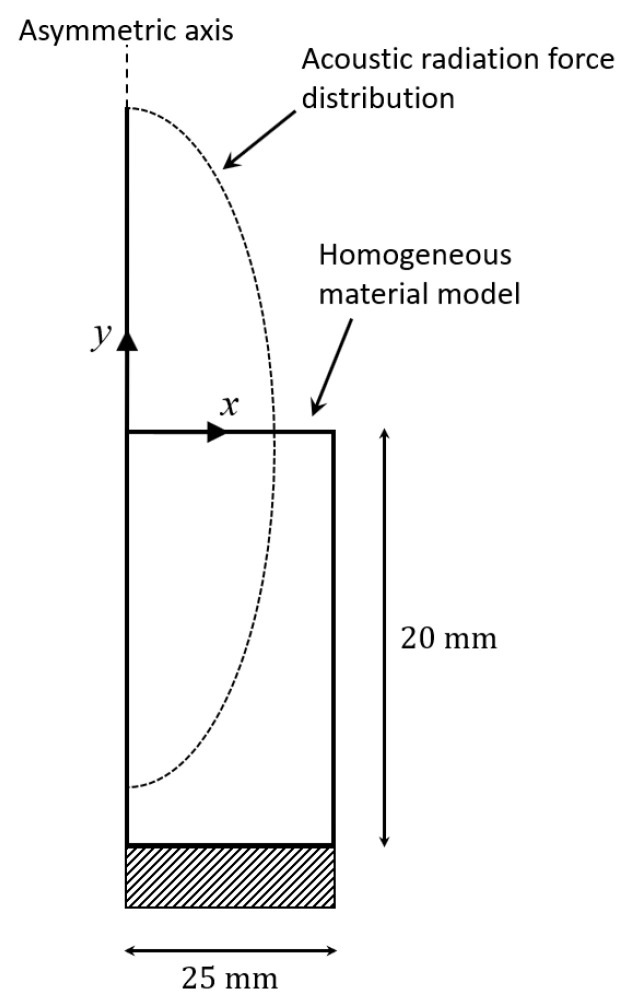
Illustration of the finite element model.

FE computer simulation was used to test the validity of RC viscoelastography. FE simulation was performed using ABAQUS/CAE 2019 (Dassault Systems Simulia Corp., Johnson, RI, USA) on an asymmetric, homogeneous FE model (Figure 4). The radius of the cross-sectional area and thickness of the model were 2.5 mm and 5 mm, respectively. The model was meshed by quadrilateral elements of dimensions 0.05 mm × 0.05 mm and comprised of 5000 elements and 5151 nodes, determined using a mesh convergence study. The setting of the boundary condition of the model was that the top and side were not constrained while the bottom was constrained along the depth direction. The center of the transducer was assumed to be aligned with the asymmetric axis of the model.

The model was constructed by an isotropic linearly viscoelastic material. Four parameters were used to define the mechanical properties of the material, including the modulus of elasticity, Poisson’s ratio, and the two parameters g and $\tau_{R}$ in the one-branch Prony series of the dimensionless relaxation modulus for defining the viscoelastic properties:

(7) $$g_{R}\left(t\right)=1-g\left(1-e^{-\frac{1}{\tau_{R}}t}\right)$$

where g is the same as the one in Equation (2), and $\tau_{R}$ is the relaxation time constant. The associated $\tau_{C}$ can be calculated by Equation (2).

The material for constructing the model was assumed to be incompressible, therefore Poisson’s ratio was set as 0.495 (the maximum Poisson’s ratio value that can be set in ABAQUS/CAE 2019).

#### 2.3.2. Setting of the Acoustic Radiation Force

The acoustic radiation force distribution was modeled by the Gaussian function applied along the depth direction. It has been suggested that the Gaussian function can adequately simulate the main properties of the acoustic radiation force distribution, since the focal zone of the acoustic radiation force—a body force as a function of space—is a Gaussian-shaped beam [36,37]. This method has been adopted by some previous studies [37,38,39]. The Gaussian function used in the present study was

(8) $$f\left(x,y\right)=F_{M}e^{-\frac{{\left(x-x_{0}\right)}^{2}}{2\beta_{x}{}^{2}}-\frac{{\left(y-y_{0}\right)}^{2}}{2\beta_{y}{}^{2}}}$$

where $x_{0}$ and $y_{0}$ are the coordinates of the center of the Gaussian function along the x (dimension along the lateral direction, of unit m) and y (dimension along the depth direction, of unit m) directions, respectively. In the present study, the acoustic radiation force was set to be focused at the origin of the model (i.e., the intersection of the axisymmetric axis and the top surface of the model); therefore, $x_{0}$ and $y_{0}$ were zero. $\beta_{x}$ and $\beta_{y}$ are associated with the −6 dB beamwidths along the x and y directions, respectively. The association between β and −6 dB beamwidth was

(9) $$-6\;\mathrm{dB}\;\mathrm{beamwidth}=2\sqrt{2\ln4}\beta$$

$F_{M}$ in Equation (8) is the maximum amplitude of the acoustic radiation force (of unit N/m^3^) at the center of the acoustic radiation force distribution, calculated by

(10) $$F_{M}=\frac{2\alpha I}{c}$$

α in Equation (10) is the absorption coefficient of the material (of unit N_p_/m) calculated by

(11) $$\alpha =\frac{1}{20}\cdot \ln\left(10\right)\cdot 100\cdot \alpha_{dB}\cdot f$$

where $\alpha_{dB}$ is the absorption coefficient of the material (of unit dB/cm/MHz), and *f* is the operating frequency of the transducer (of unit MHz). In the present study, the $\alpha_{dB}$ of muscle tissue was used, designated as 0.57 according to Hoskins et al. [40].

I in Equation (10) is the temporal average intensity (of unit W/m^2^) calculated by

(12) $$I=\frac{p^{2}}{2\rho c}$$

where p is the amplitude of the acoustic pressure, and ρ is the density of the material, set as 1000 kg/m^3^. c in Equations (10) and (12) is the compressional sound speed of the material, set as 1540 m/s.

In the simulation, the time duration for initiating the acoustic radiation force was designed to be short (500 µs) in order to simulate a step excitation [41]. $F_{M}$ was then increased linearly in the ramp mode, or kept constant in the creep mode, until the strain response of each element of the model reached the steady state. The time for the strain response to reach the steady state could be different in different modes and could also depend on the two parameters relevant to the viscoelastic properties g and $\tau_{R}$. In the present study, the simulation time was set as a constant of 500 s in both modes.

The acoustic radiation force was assumed to be applied by a transducer having the center frequency of 2 MHz (H148; Sonic Concepts, Woodinville, WA, USA; −6 dB beamwidth of 1.5 mm along the lateral direction; −6 dB beamwidth of 8.0 mm along the depth direction). The associated $\beta_{x}$, $\beta_{y}$, and α were 4.5042 × 10^−4^, 0.0024, and 13.1247 N_p_/m, respectively, according to Equations (9) and (11). In the ramp mode, p was set as a constant of 2 MPa, and the operating frequency of the transducer was gradually increased linearly from 1.95 to 2 MHz (at a rate of 100 Hz/s) such that $F_{M}$ was increased linearly from 21,583 to 22,137 N/m^3^ (at a rate of 1.107 N/m^3^/s). In the creep mode, p was set as a constant of 2 MPa and the operating frequency was set as a constant of 2 MHz, and therefore the associated $F_{M}$ was fixed at 22,137 N/m^3^. It must be noted that the setting of frequency range is dependent on the choice of transducer, and the description above represents the specific settings used in the present study.

Focused ultrasound pulses were assumed to be applied with 1 Hz pulse repetition frequency and 0.99 s pulse duration (or 99% duty cycle). Because of this high duty cycle of the focused ultrasound pulse, the acoustic radiation force was assumed to be applied continuously during the simulation, ignoring the transient effects associated with turning the pulse ON and OFF [42].

#### 2.3.3. Data Analysis

**Figure 5:**
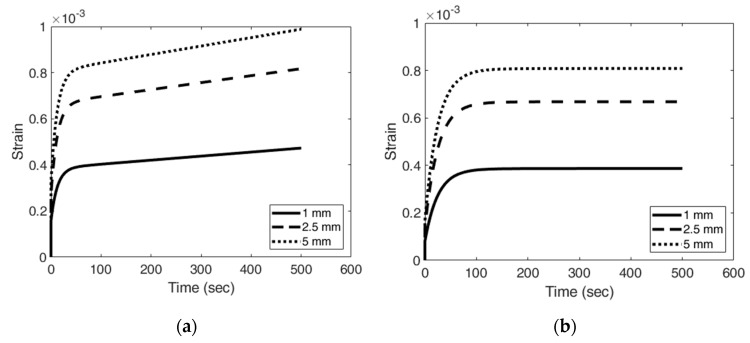
The strain responses over time at different depths along the asymmetric axis of the model in the ramp mode (**a**) and creep mode (**b**).

The strain over time of each element of the model was calculated in each mode, and then imported into MATLAB (R2019a; Mathworks, Natick, MA, USA) for further analysis. Figure 5a shows an example of the strain responses over time at different depths along the asymmetric axis of the model in ramp mode. Figure 5b shows the responses in creep mode. The three viscoelastic parameters of the material ($\tau_{R}$, $\tau_{C},$ and g) could be quantitatively evaluated from the strain over time data in the two modes (please see the Section 2.2 for the details and Section 2.2.4 for the summary).

The image for illustrating the distribution (or map) of each parameter could be produced by collecting the value of the parameter of each element in the model. The image was mirrored to produce a full two-dimensional map, since the FE model was axisymmetric.

In the simulation, two groups of materials were studied. In the first group, $\tau_{R}$ = 0.5, 2, 5, and 10 s while the modulus of elasticity = 10 kPa and g = 0.8. In the second group, g = 0.4, 0.6, 0.8 and 0.9 while the modulus of elasticity = 10 kPa and $\tau_{R}$ = 5 s.

In each group, the value of each parameter obtained from the simulation (the median value of the image) was compared to the theoretical value set in ABAQUS for testing the validity of RC viscoelastography. If they are comparable, it can be claimed that the design of RC viscoelastography and the relevant principle are valid.

## 3. Results

**Figure 6:**
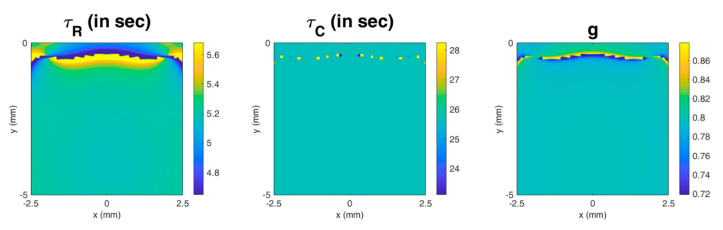
The image for each parameter in a case (the modulus of elasticity = 10 kPa, $\tau_{R}$ = 5 s, and g = 0.8). It can be observed that the image for each parameter is relatively homogeneous, except for the region near the top surface of the material.

The image for each parameter in one case (modulus of elasticity = 10 kPa, $\tau_{R}$ = 5 s, and g = 0.8) is shown in Figure 6. Since the pattern of the image for each parameter is similar in all cases, only one set of images in a given case is demonstrated. It can be observed that the image for each parameter is relatively homogeneous, except that there are artifacts near the top surface of the material. The artifacts occur at depths shallower than approximately 1 mm, 0.7 mm, and 0.8 mm for $\tau_{R}$, $\tau_{C}$, and g, respectively.

Table 1 shows the comparison between the value of each parameter obtained from the simulation and the corresponding theoretical value set in ABAQUS. It can be observed that the simulation value is very close to the theoretical one for all three parameters. For the two viscoelastic time constants, the error for $\tau_{R}$ is generally larger than that for $\tau_{C}$, except for the first case in the second group; in all of the cases examined, the error is smaller than 5%, except for the error for $\tau_{R}$ (6.58%) for the fourth case in the second group. For g, the error is smaller than 0.33% in each case. In summary, the measurement accuracy for g is the highest and much higher than that for $\tau_{C}$, which in turn is slightly higher than that for $\tau_{R}$.

It is interesting to note that the errors for $\tau_{R}$ and $\tau_{C}$ decrease with increasing theoretical value of $\tau_{R}$ in the first group, while they increase with increasing theoretical value of g in the second group. The error for $\tau_{R}$ becomes significant (higher than 5%) for the fourth case in the second group when g is 0.9. In [35], it was found that the error of the estimation of viscoelastic time constants increased nonlinearly with increasing g, and became larger and larger when g was larger than 0.9 and approached 1. The possible reason for this phenomenon is that numerical divergence can occur and get larger when g approaches its upper limit 1, causing the distortion of the simulated strain–time curve and resulting in a significant error.

The results suggest that RC viscoelastography could generally evaluate the viscoelastic parameters of the material with good accuracy, including $\tau_{R}$, $\tau_{C}$, and g, except for the region near the top surface of the material.

## 4. Discussion

The present study proposes a viscoelastography method based on ultrasound acoustic radiation force, named RC viscoelastography, for the noninvasive measurement of the parameters relevant to the viscoelastic properties of a material. FE computer simulation demonstrated that RC viscoelastography can provide accurate measurement, justifying the validity of the principle on which the proposed method is based. The proposed method could have several potential applications. First, it could be used as a tool for evaluating the mechanical properties of biomaterials during the development process. In tissue engineering, biomaterial scaffolds are used to mimic the properties of the extracellular matrix in order to optimize tissue regeneration [30]. The mechanical properties of scaffolds have significant effects on cell behaviors including cell attachment, proliferation, and differentiation [43], and play an important role in determining the environment for the development of cells and biomaterials [44]. Hence, it is necessary to have noninvasive methods to evaluate the mechanical properties of biomaterials during development in order to ensure their quality [9]. Second, the technique could be used to evaluate the mechanical properties of materials for implants during design. Materials for implants should have proper mechanical properties for adequate support, stabilization, and flexibility [45], such that the implant can emulate the mechanical function of the native tissue it replaces and can drive the formation of new tissues [46,47]. Third, the proposed method could be used to diagnose diseases of organs in vivo, such as liver fibrosis or breast lesions [48]. However, since the proposed method may need a relatively longer period of time to apply acoustic radiation force excitations during the measurement, temperature rise is an important problem that should be carefully investigated before the proposed method is used for biological tissues in vivo.

This study found that the proposed RC viscoelastography method was able to accurately evaluate the relaxation and creep time constants of materials. Several studies have shown that the viscoelastic time constant can be used as a biomarker for the quantitative diagnosis of pathological conditions of tissues [49,50,51], or as an indicator for quantitative evaluation of the status of biomaterials during the development process [30,31]. Hence, although RC viscoelastography cannot evaluate the modulus of elasticity, it could still be a useful diagnosis or evaluation tool based on quantifying the viscoelastic time constants.

Though RC viscoelastography can accurately evaluate viscoelastic parameters including the relaxation time constant ($\tau_{R}$), creep time constant ($\tau_{C}$), and the ratio of viscous fluids to solids in a material (g), it cannot evaluate the modulus of elasticity, which is an important mechanical property reflecting the stiffness of the material. The lack of information about the magnitude of stress within the material is the reason why the proposed method cannot evaluate the modulus of elasticity, since both stress and strain are needed to evaluate this property, according to the principle of mechanics. Below, a possible strategy that could be used to solve this problem is proposed. Based on the principle in the present study, if r can be obtained, the modulus of elasticity ($E_{1}$) can be obtained, since $r/E_{1}$ can be obtained directly from experimental data. The problem is that r is the rate of increase of the stress of an element within the material in ramp mode, and cannot be obtained experimentally. The rate of increase of the acoustic radiation force is another value that cannot be obtained. Fortunately, they are directly proportional to the linear rate of increase of the operating frequency of the transducer, which is known and controllable. Based on this fact, it would be possible to develop a model to estimate r where the linear rate of increase of the operating frequency is used as the input, using some assumptions and the principle of continuum mechanics. In other words, the relationship between r and the linear rate of increase of the operating frequency could be found and used to calculate r. However, if this idea can be successfully implemented and incorporated into the proposed method in the future, errors will be introduced because the idea relies on calculation based on assumptions and modeling, and assumptions and modeling inevitably bring errors to a certain extent. This is an interesting and important topic to be investigated in the future.

The novelty of RC viscoelastography is that there is no need to know the magnitude of acoustic radiation force and induced stress in the material in order to evaluate the viscoelastic parameters of the material, and all information needed to evaluate viscoelastic parameters can be directly obtained from experimental data. No other models or assumptions are needed. In the existing acoustic radiation force creep imaging methods, the stress induced by the acoustic radiation force is a required input for fitting the measured curve in order to obtain the viscoelastic properties [52,53]. For example, in the method developed by Mauldin et al. [29], many parameters need to be determined in advance by fundamental experiments in order to estimate the spatial distribution and magnitude of the acoustic radiation force and induced stress. Similarly, in another study by Hong et al., a specific experimental design is needed in order to determine the absorption coefficient and sound speed of the material, acoustic intensity at the chosen location, and focused ultrasound beam profile in water, and then these are used to calculate the stress value induced by the acoustic radiation force at the chosen location. The calculated stress value is then used as the input for the curve-fitting process. Obviously, some assumptions are needed for this calculation. In the present study, our proposed RC viscoelastography uses two ultrasound excitation modes to excite the material. By combining and analyzing the information obtained from these two modes of excitation, no assumptions are required, and the knowledge regarding the distribution and magnitude of the acoustic radiation force is not needed for evaluating the viscoelastic parameters. However, as mentioned previously, RC viscoelastography cannot evaluate the modulus of elasticity, although it can accurately evaluate viscoelastic parameters including $\tau_{R}$, $\tau_{C}$, and g. The modulus of elasticity is a very important mechanical property reflecting the stiffness of the material, and needs to be determined for certain applications. In the future, it is necessary to develop a force-independent ultrasound method that can completely evaluate all of the viscoelastic properties, including the modulus of elasticity.

**Figure 7:**
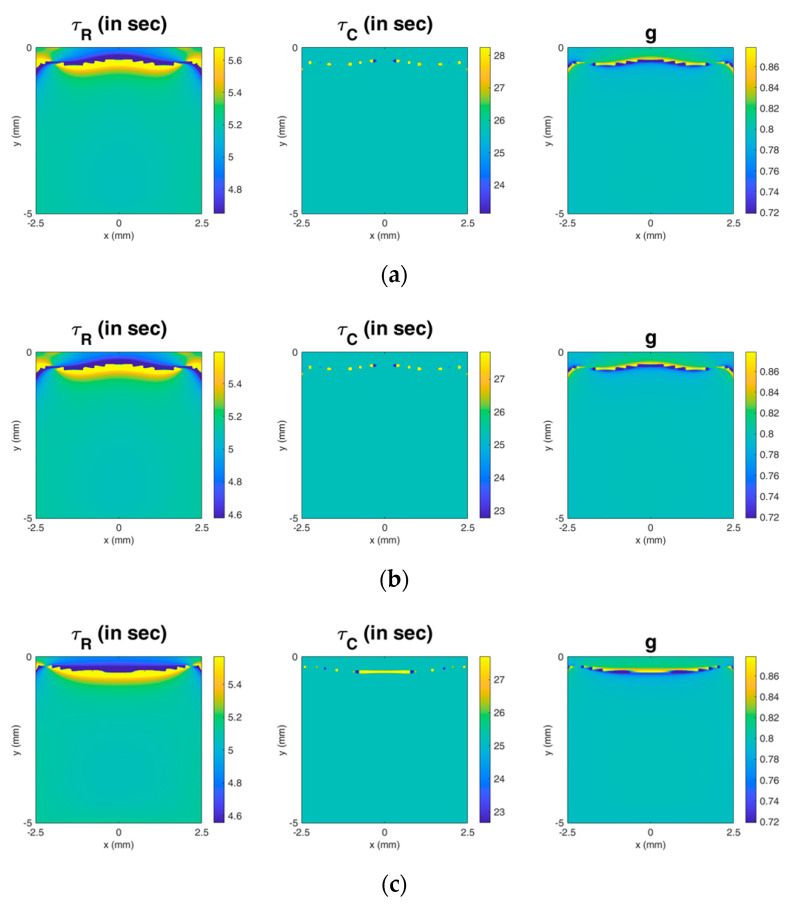

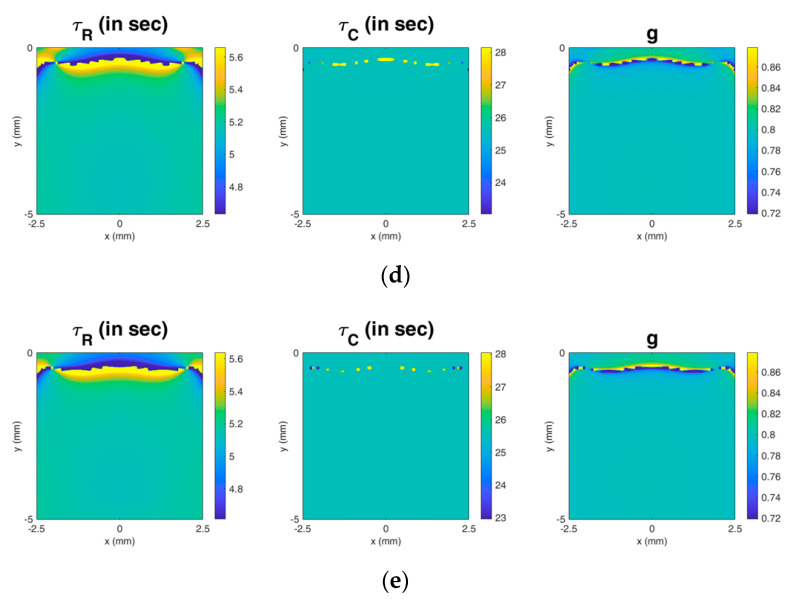
The effect of the selected ultrasound parameters on the pattern of the artifact. In these simulations, the modulus of elasticity, $\tau_{R}$, and g were set as 10 kPa, 5 s, and 0.8, respectively. (**a**) Default setting as described in the main text. (**b**) The same as the default, except that the center frequency of the transducer is 2 MHz. (**c**) The same as the default, except that the transducer has a −6 dB beamwidth of 1.5 mm along the lateral direction and a −6 dB beamwidth of 8.0 mm along the depth direction. (**d**) The same as the default, except that the location of the focus of the acoustic radiation force excitation is ($x_{0}$, $y_{0}$) = (0 mm, 0.5 mm). (**e**) The same as the default, except that the location of the focus of the acoustic radiation force excitation is ($x_{0}$, $y_{0}$) = (0 mm, −0.5 mm).

Based on the simulation results, it can be observed that RC viscoelastography cannot provide an accurate measurement for the region near the top surface of the material; there are significant artifacts near the top surface of the material. The artifacts are probably caused by complex boundary conditions on the top surface of the material due to the nature of the acoustic radiation force excitation. In addition, the pattern of the artifact could depend on ultrasound parameters. For example, as shown in Figure 7, a set of simulation results shows that the pattern of the artifact is dependent on the beamwidth of the transducer and the location of the focus of the acoustic radiation force excitation, but is not dependent on the center frequency of the transducer that determines the magnitude of the acoustic radiation force. Hence, based on this preliminary finding, parameters related to the spatial distribution of the acoustic radiation force could affect the pattern of the artifact, but the difference is very insignificant. On the other hand, parameters related to the magnitude of the acoustic radiation force might not have an effect on the pattern of the artifact. Based on the current simulation results, the artifact occurs at depths shallower than approximately 1, 0.7, and 0.8 mm in the images of $\tau_{R}$, $\tau_{C}$, and g, respectively. Considering that there will be more or less noise in a real experiment that may further affect the distribution of the artifact, I suggest that it would generally be better to select an image area deeper than 1 mm. However, this suggestion is based on the settings of the present study, and should not be regarded as the most general one. This important topic must be investigated in detail in the future, and the findings could offer a more general suggestion about guiding the selection of the image area.

**Figure 8:**
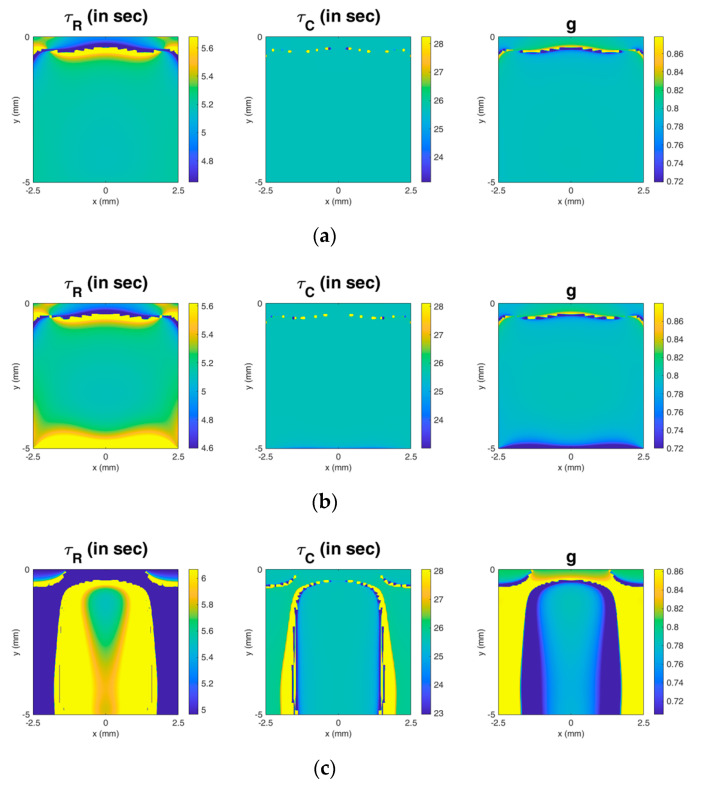
The effect of the type of boundary conditions on the measurement results. In the images, the region providing accurate measurement results shows turquoise color. (**a**) The bottom is constrained along the depth direction while the side and top are not constrained. (**b**) The bottom is constrained along all directions while the side and top are not constrained. (**c**) The bottom is constrained along the depth direction and the side is constrained along the lateral direction, while the top is not constrained.

In addition to transducer parameters, the type of boundary conditions (related to the method of securing the sample in a real experiment) does have an impact on the region providing accurate measurements in the image. For example, as shown in Figure 8b, if the bottom is constrained in all directions while the side and top are not constrained (meaning that the method for securing only secures the bottom of the sample and has minimal contact with the sides), there will be errors in a small region near the bottom. However, as shown in Figure 8c, if the bottom is constrained along the depth direction and the side is constrained along the lateral direction while the top is not constrained (i.e., the sample is put into and secured by a box holder), the region providing accurate measurements becomes much smaller. This implies that in a real experiment, the sample should not be secured using a box holder. Inevitably, a method for securing the sample is necessary. The simulation results can help to understand the effect of the type of boundary conditions and to guide the optimal experimental design.

In addition to the limitations mentioned above, there are other limitations to the proposed method: (1) In this first step of the development of RC viscoelastography, FE computer simulation was used to validate the relevant principle and performance of this newly proposed technology. In the future, experiments are necessary to test the performance of RC viscoelastography on real biomaterials and biological tissues; (2) The principle for developing RC viscoelastography is based on the standard linear solid model, which is one of the simplest and most fundamental models in linear viscoelasticity for modeling materials having simple microstructures. Hence, it is unclear whether RC viscoelastography can accurately evaluate the viscoelastic parameters of materials having more complex microstructures (e.g., real biomaterials and biological tissues). Experiments are needed in order to investigate this topic in the future; (3) RC viscoelastography cannot provide a real-time measurement, since the steady-state responses of both ramp and creep modes are necessary in order to obtain the required data. The time period for reaching the steady-state response in each mode depends on the intrinsic mechanical properties of the material. In the future, it is important to study how much temperature will rise due to this significant time period of focused ultrasound excitation; (4) Only homogeneous materials were investigated in the present study. The performance of RC viscoelastography for measuring heterogeneous materials should be investigated in the future.

## 5. Conclusions

This paper introduced a novel viscoelastography method based on ultrasound acoustic radiation force, named RC viscoelastography, for noninvasive measurement of the viscoelastic parameters of materials. FE computer simulation demonstrated that RC viscoelastography can accurately evaluate the viscoelastic parameters of the material, including the relaxation and creep time constants and the ratio of viscous fluids to solids in the material, except for the region near the top surface of the material. The novelty of RC viscoelastography is that there is no need to know the magnitude of acoustic radiation force or induced stress in the material in order to evaluate the viscoelastic parameters. The main limitation of RC viscoelastography is that it cannot evaluate the modulus of elasticity. In the future, it is necessary to improve the RC viscoelastography technology or to develop a force-independent ultrasound method that can completely evaluate all of the viscoelastic properties, including the modulus of elasticity. In addition, experiments are necessary to test the performance of RC viscoelastography in real biomaterials and biological tissues.

## Figures and Tables

**Table 1:** Comparison between the values of properties set in ABAQUS and the ones obtained from the simulation.

	Theoretical Properties			Properties Obtained from the Simulation			Error (%)		
Material Number	$\tau_{R}$	$\tau_{C}$	g	$\tau_{R}$	$\tau_{C}$	g	$\tau_{R}$	$\tau_{C}$	g
**Group 1**									
**1**	0.5	2.5	0.8	0.523	2.584	0.798	4.60	3.36	0.25
**2**	2	10	0.8	2.091	10.336	0.798	4.55	3.36	0.25
**3**	5	25	0.8	5.196	25.690	0.798	3.92	2.76	0.25
**4**	10	50	0.8	10.375	51.295	0.798	3.75	2.59	0.25
**Group 2**									
**1**	5	8.3	0.4	5.060	8.418	0.399	1.2	1.42	0.25
**2**	5	12.5	0.6	5.093	12.678	0.598	1.86	1.42	0.33
**3**	5	25	0.8	5.196	25.690	0.798	3.92	2.76	0.25
**4**	5	50	0.9	5.329	51.941	0.897	6.58	3.88	0.33
